# Supplementary material for: Chemical compass behaviour at microtesla magnetic fields strengthens the radical pair hypothesis of avian magnetoreception
Source: Nat Commun. 2019 Aug 16;10:3707. doi: 10.1038/s41467-019-11655-2 (PMC6697675; doi:10.1038/s41467-019-11655-2)
Supplement: Supplementary file 1 — Supplementary Information [file 41467_2019_11655_MOESM1_ESM.pdf]

Supplementary Information for

Chemical compass behaviour at microtesla magnetic fields strengthens the radical pair hypothesis of avian magnetoreception

Kerpal et al.

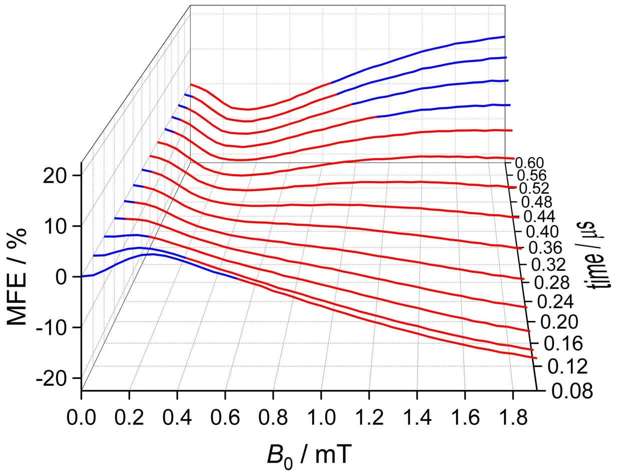

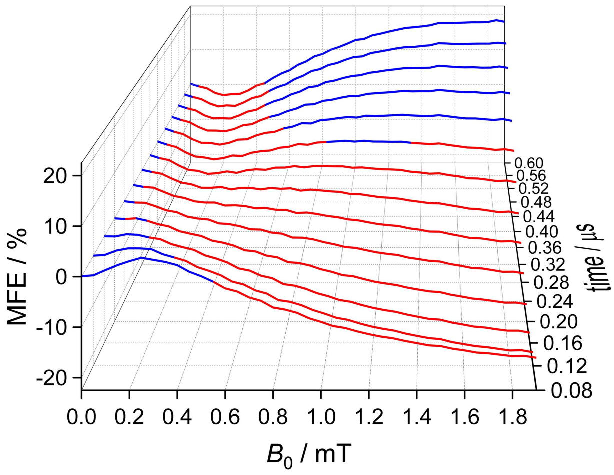

Supplementary Figure 1: Time and field dependence of the magnetic field effect (MFE). Percentage MFE for **CPF** (*left*) and **CPF$_\mathbf{D}$** (*right*) as a function of the applied magnetic field for different times after laser excitation. The data shown have been averaged over a time window of 20 ns centred around the indicated times. Positive MFE values are shown in blue, whereas negative values are shown in red. It can be seen that the low field MFE changes its sign significantly earlier than the high field MFE.

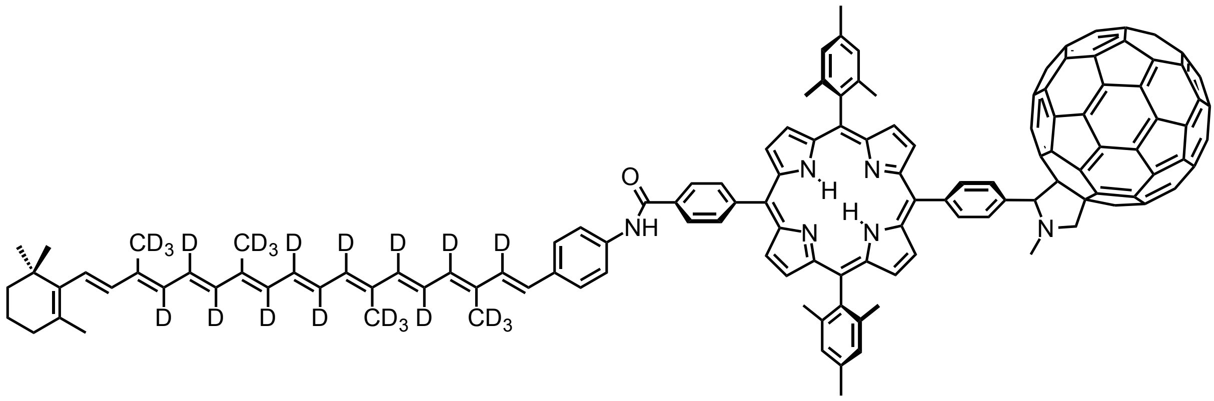

Supplementary Figure 2: Chemical structure of the deuterated triad **CPF$_\mathbf{D}$**.

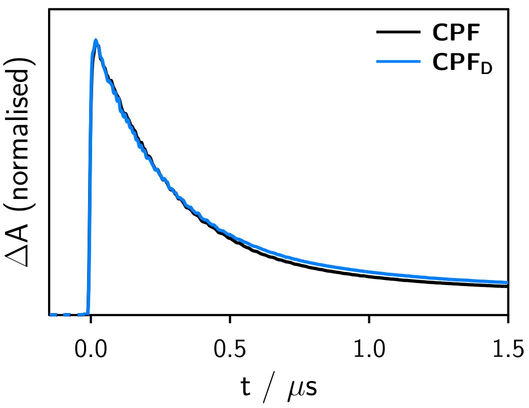

Supplementary Figure 3: Comparison of the kinetics of the protonated and deuterated **CPF** triads in the absence of a magnetic field.

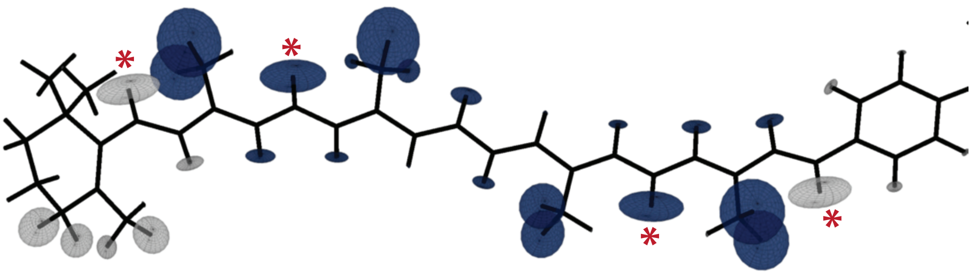

Supplementary Figure 4: Visualisation of the calculated (B3LYP/EPRII) proton hyperfine coupling tensors in the carotenoid radical. The relative size of the tensors reflects the magnitude of the hyperfine coupling in the different directions. The tensors shown in dark blue correspond to the protons replaced by deuterium in $\mathbf{CPF_D}$. It can be concluded that the anisotropic field response mainly stems from four protons (hyperfine tensors marked with a red asterisk).

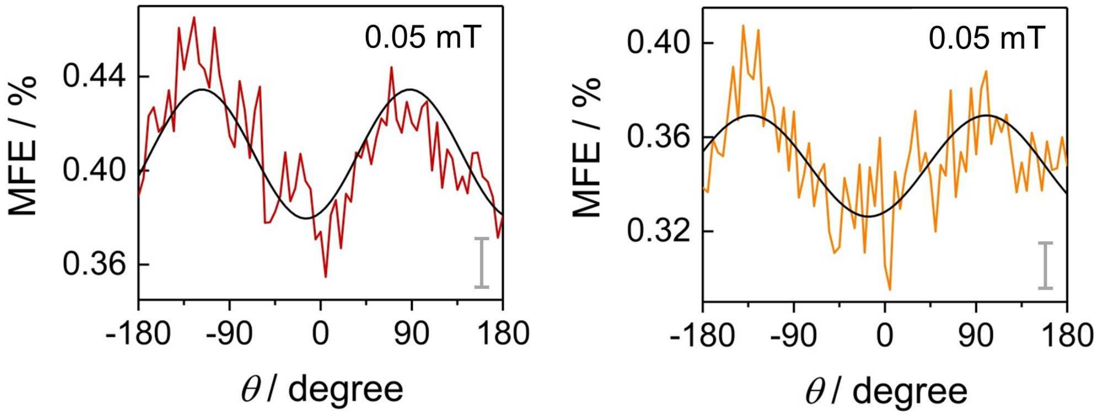

Supplementary Figure 5: Orientation dependence of the magnetic field effect (MFE) at 0.05 mT. MFE as a function of θ for **CPF** (*left*) and $\mathbf{CPF_D}$ (*right*) recorded at the approximate average of the Earth's field (0.05 mT). The polarisation directions of both pump and probe beams were parallel to the laboratory *z*-axis and the data shown were averaged over a time window from 0.07 μs to 0.09 μs after laser excitation. The grey bars indicate the (average) standard deviation of the mean. The black solid lines represent the best sinusoidal fits to the data. Measurements at this field strength test the limits of our experimental apparatus as evident by the reduced signal-to-noise levels and the reduced invariance to an inversion of the field (compared to the data shown in the main manuscript). However, the observed angular dependence can still be fit satisfactorily with a sinusoidal function and the observed phase is, as expected, entirely consistent with that observed for higher field strengths.

Supplementary Note 1: Magnetic Field Calibration

In the experiment, the sample is held at the centre of three pairs of Helmholtz coils, which are aligned along the x, y and z-axes of the laboratory frame. The two main pairs of coils generate fields of up to 2 mT along the x and z axes. Each pair of coils in independently driven by a computer-controlled power supply, allowing fields to be produced in an arbitrary direction in the x/z-plane. These fields are characterised by a field strength, B and a field angle, θ, measured from the $-z$-axis (cf. Figure 2 in the main manuscript). The field coils have to be calibrated so that the desired field strength and angle can be set accurately. The calibration procedure for the main coils in x and z-direction is presented below.

Any residual fields in the x and z directions, including the Earth's magnetic field, can be cancelled by calibration of the main coils. The remaining component along the y-axis cannot be cancelled using these coils alone. Therefore, a third pair of coils was installed, aligned along the y-axis. These coils can produce a field of up to 0.2 mT, allowing the component of the Earth's magnetic field along this axis to be cancelled. They are not under computer control; the field produced is pre-set to a fixed value which cancels the y-component of any residual field. The value is found by measuring the magnetic field along the y-axis at the sample location, using a sensitive Gauss-meter and adjusting the strength of the field produced by the shim coils in y-direction until no residual field remains.

Calibration procedure. The magnetic field produced by the coils is changed by sending two digital control values from the instrument control computer to the power supply for each pair of coils. The field produced depends on these control values

$$B_x = f\left(\mathrm{CV}_1, \mathrm{CV}_2\right) \tag{1}$$

$$B_z = g\left(\mathrm{CV}_1, \mathrm{CV}_2\right) \tag{2}$$

Here, B_x and B_z are the field strengths in the x and z-directions, and CV_1 and CV_2 are the two control values sent to the power supply. f and g are two functions which link the control values to the magnetic field produced at the sample. Calibration entails finding the inverse functions,

$$\mathrm{CV}_1 = f'\left(B_x, B_z\right) \tag{3}$$

$$\mathrm{CV}_2 = g'\left(B_x, B_z\right) \tag{4}$$

allowing the field at the sample to be set.

First, a calibration table is generated by measuring the field strengths, B_x and B_z, for a number of (CV_1, CV_2) pairs with the active part of the Hall probe fixed exactly at the sample location.* To set the field to a particular combination of B_x and B_z, the point on both surfaces which corresponds to those B_x and B_z values is found. The precise values of CV_1 and CV_2 which will produce the desired field are found by interpolating between the points in the calibration table. These values are then sent to the magnetic field power supply.

To increase our field resolution, the 1024 possible values for CV_1 and CV_2 were mapped on a range from -0.5 mT to $+0.5$ mT for the data shown in Figure 2a in the main text and Supplementary Figure 4, rather than on the complete accessible range from -2 mT to $+2$ mT.

*Only a small subset of the possible (CV_1, CV_2) pairs can be measured: The digital control values, CV_1 and CV_2, can each take one of 1024 possible values. Therefore, there are over 10^6 (CV_1, CV_2) pairs.

Supplementary Note 2: Experimental Setup

A schematic drawing of the experimental setup, based on a standard transient absorption experiment, is shown below. The sample is excited with linearly polarised pulsed laser light at 532 nm at a repetition rate of 10 Hz (Nd:YAG, Continuum Minilite) using pulse energies of < 1 mJ. A cw laser diode with a central wavelength of 980 nm is used as the probe light source. To prevent excessive sample heating, the probe light is only switched on during a narrow time window needed for data acquisition (4 % duty cycle, $\sim$1.5 mW measured cw power).

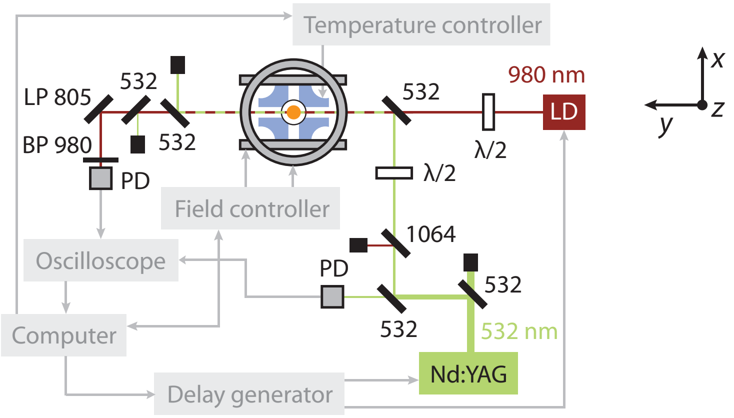

Supplementary Figure 6: Schematic drawing of the experimental setup.

The polarisation of the pump and probe beams are controlled by means of two half-wave plates and are chosen to be identical in every experiment to facilitate the analysis of the anisotropy data. The polarisation of the laser light after the half-wave plate was verified using a Glan-Taylor polariser. Special care was taken to keep the laser beam alignment perfectly horizontal.

The pump and probe beams (diameter roughly 3 mm) are collinear at the sample to ensure maximum overlap and dielectric mirrors are used to couple the pump beam in and out of the probe light path. In addition, two dielectric long pass filters are used to eliminate residual pump light scatter.

The sample is sitting in a liquid nitrogen flow cryostat (Oxford Instruments) which allows to maintain a constant sample temperature. The cryostat is surrounded by three pairs of orthogonal Helmholtz coils. The coils in x and z direction, with approximate diameters of 16 and 20 cm, respectively, are carefully calibrated to precisely control the magnetic field strength and field angle (cf. Supplementary Note 1). The third pair of coils (diameter $\sim$7 cm) is set to a constant offset and serves to cancel out the component of the Earth magnetic field in propagation direction (y).
